# Animal-free safety assessment of chemicals: an innovation system perspective

**DOI:** 10.1007/s00204-024-03878-0

**Published:** 2024-10-04

**Authors:** Marjolein J. Hoogstraaten, Jelle Vriend, Victoria C. de Leeuw, Simona O. Negro, Ellen H. M. Moors, Anne S. Kienhuis, Jarno Hoekman

**Affiliations:** 1 Copernicus Institute of Sustainable Development, Utrecht University, Utrecht, The Netherlands; 2 National Institute for Public Health and the Environment (RIVM), Bilthoven, The Netherlands; 3 Breakthrough Tech Innovation Group, Vrije Universiteit Amsterdam (VU), De Boelelaan 1108, 1081 HZ Amsterdam, The Netherlands; 4 Institute for Risk Assessment Sciences, Utrecht University, Utrecht, The Netherlands

**Keywords:** Innovation systems, Animal-free chemical safety assessment, Transitions, Toxicology, New approach methodologies

## Abstract

This perspective paper, which is the result of a collaborative effort between toxicologists and scholars in innovation and transition studies, presents a heuristic framework based on innovation system literature for understanding and appraising mission achievement to animal-free chemical safety assessment using New Approach Methodologies (NAMs). While scientific and technical challenges in this area are relatively well known, the recent establishment of missions and roadmaps to accelerate the acceptance and effective use of NAMs for chemical safety assessment raises new questions about how we can grasp the systemic nature of all changes needed in this transition. This includes recognising broader societal, institutional, and regulatory shifts necessary for NAM acceptance and uptake. Our paper discusses how the innovation system approach offers insights into key processes and associated activities that include as well as transcend the technical and scientific realm, and can help to accelerate acceptance and uptake of NAMs. Based on these insights, we present a comprehensive framework that, next to scientific and technological developments, recognises the need for coordinated efforts in areas like education, training, funding, policy-making, and public engagement to promote the acceptance and uptake of NAMs. Our framework can be used to perform structural and functional analyses of the innovation system of NAMs and animal-free safety assessment and as such provides handholds to track progress and organise collective efforts of actors to make sure we are moving in the right direction.

**Supplementary Information:**

The online version contains supplementary material available at 10.1007/s00204-024-03878-0.

## Introduction

Human health safety assessment of chemicals is traditionally performed using animal studies, which are still considered to be ‘the gold standard’ by both industry and regulators (Sewell et al. 2017; Westmoreland et al. 2022). Efforts are increasingly being made to innovate human health safety assessment with the aim to no longer rely on animal testing and move towards a more human relevant safety assessment approach. These efforts attempt to benefit animal welfare and improve the predictive value of methods for the purpose of human health safety assessment (Punt et al. 2011; Westmoreland et al. 2022). Collectively, these innovative methods are referred to as New Approach Methodologies (NAMs) and comprise novel technologies, methodologies, approaches, or combinations thereof that provide information on chemical safety (ECHA 2016; US EPA 2018). Stimulating the acceptance of NAMs by a broad range of stakeholders is a topic addressed in various roadmaps on the transition towards non-animal testing in human health safety assessment (NCad 2016; RIVM 2018; ICCVAM 2018, 2024; EFSA 2021; European Commission 2023a, b).

However, substantial investment in NAM development and their significant role in missions and roadmaps towards animal-free safety assessment of chemicals have not yet resulted in the desired reduction in the number of animals used for safety assessment (Ball et al. 2022). One reason is that in order for NAMs to make the step to widespread acceptance and effective use, interventions that operate at the system level are needed (Mathisen et al. 2024). These interventions include broader social and institutional (we understand institutions as humanly devised constraints that structure political, economic and social interactions (North 1991: 97)) shifts in mindsets, regulatory frameworks, and congruent risk-assessment practices, next to scientific and technological change. For instance, rather than using toxicological endpoints in traditional animal studies as the basis for hazard identification and safety assessment, several research projects—such as the VHP4Safety project (www.vhp4safety.nl, NWA-ORC 1292.19.272)—advocate a mindset shift towards understanding human biology and physiology as the backbone for specific human disease endpoints. Central in this approach is the development of adverse outcome pathways (AOPs) which requires integration of insights from different disciplines. Others have also advocated for a broad shift in other “focus areas” such as open science and data sharing, targeted funding for NAM development and uptake, interdisciplinary education, and facilitation of a shift in societal views on animal-free safety assessment (Abarkan et al. 2022).

The recognition of the need for broader shifts underscores the systemic nature of the processes of developing, diffusing, and implementing solutions such as NAMs, intended to achieve missions towards animal-free safety assessment. Because of this systemic nature, scholars in the field of innovation and transition studies argue that employing an innovation system approach—and specifically constructing a comprehensive framework for NAM acceptance and uptake based on this approach—can enhance the understanding, coordination, and evaluation of processes that accelerate the development and uptake of new innovations such as NAMs (Elzinga et al. 2023; Hekkert et al. 2020). The innovation system approach focuses on the varied activities and interactions amongst actors that are required for the development, diffusion, and implementation of innovations, as well as the governance processes to structure, coordinate, and align these activities. This approach is particularly helpful to heuristically distinguish between different key processes that together shape the functioning of an innovation system that contributes to mission achievement.

For instance, to achieve the mission towards animal-free safety assessment, the innovation system needs to function well in terms of the demanding process of regulatory acceptance of various types of NAMs (Schiffelers et al. 2012), which is a frequently cited barrier for NAM implementation. Simultaneously, the innovation system needs to build capabilities for innovating safety assessment through education and training activities (Abarkan et al. 2022) and facilitate network formation between public and private actors, amongst others. Such key processes can then reinforce each other creating positive feedback loops between them which contribute to mission progress and achievement. In contrast, a diagnosis of negative feedback loops between processes can inform options for interventions in the system by for instance policy-makers.

The aim of this paper is to illustrate the value of employing an innovation system approach to comprehensively capture the various key processes and associated activities necessary for accelerating the acceptance and effective use of NAMs in the mission towards animal-free safety assessment of chemicals. We argue that this understanding sheds a new light on the coordination and engagement of actors to set and achieve mission goals and priorities for technological, societal, and institutional change towards animal-free safety assessment. Additionally, our effort illustrates the added value of interdisciplinary collaboration between toxicologists and social scientists in developing approaches to accelerate the acceptance and effective use of NAMs.

In this interdisciplinary collaboration, we integrated the implementation curve of NAMs that was developed by toxicologists (RIVM 2022) with the theoretical foundations of the innovation system approach developed by innovation scholars (Hekkert et al. 2007, 2020). The implementation curve of NAMs for chemical safety assessment outlines the different phases a NAM typically follows to be adopted for use in regulatory frameworks for safety assessment. The innovation system approach shows how these phases are a constitutive part of a larger set of key processes that have been described in the innovation system literature as being crucial for the development and uptake of innovations such as NAMs (Elzinga et al. 2023; Hekkert et al. 2007).

## An innovation system approach to stimulate the acceptance of NAMs for chemical safety assessment

### The landscape NAMs for safety assessment of chemical substances and the implementation curve

**Fig. 1:**
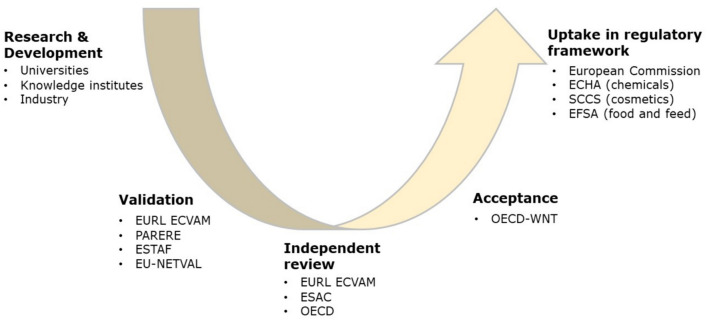
Implementation curve as developed by the Dutch National Institute for Public Health and the Environment, illustrating the scientific and technological phases of NAMs (adapted from RIVM 2022). Organisations mentioned in the figure are explained in Table 1

Recently, the Dutch National Institute for Public Health and the Environment (RIVM) developed the Landscape NAMs for safety assessment of chemical substances (RIVM 2022). This landscape describes an implementation curve consisting of the phases a NAM typically goes through to become accepted and effectively used, from research and development towards validation, acceptance, and uptake in European regulatory frameworks. In consultation with experts involved in the various phases on the curve, the RIVM mapped specific actors responsible for stimulating progress within each phase (e.g., scientists from academia that develop NAMs or risk assessors in advisory committees of the European Commission that advise on the regulatory relevance or scientific reliability of NAMs). The implementation curve is presented in Fig. 1 and a description of the various phases, and the actors involved is presented in Table 1.

**Table 1:** Overview and description of the various phases in the implementation curve

Implementation curve phase	Description	Involved actors
Research and development	In this phase, research activities by universities, research institutes and companies are conducted to develop NAMs (including in silico and in vitro testing, physiologically based kinetic (PBK) models and statistical tools to interpret data) and a better understanding of relevant underlying mechanisms using for example adverse outcome pathways (AOPs). This knowledge can then be applied to develop new testing methods and strategies	Universities, research institutes, industry
Validation	In this phase, the relevance and reproducibility of test methods for a specific purpose are evaluated to be able to set standards. Validation can occur in a prospective and retrospective manner. To increase chances of success, innovators are encouraged to follow the principles formulated by the Organisation for Economic Co-operation and Development (OECD) (series on testing and assessment) and the European Union Joint Research Centre for Alternatives to Animal Testing (EURL ECVAM)	EURL ECVAM, EURL ECVAM Network for Preliminary Assessment of Regulatory Relevance (PARERE), EURL ECVAM Stakeholder Forum (ESTAF), European Union Network of Laboratories for the Validation of Alternative Methods (EU NETVAL), EURL ECVAM Scientific Advisory Committee (ESAC), OECD
Acceptance	In this phase, a test method is formally accepted in the test guidelines programme of the OECD, generally after an independent review is conducted. The test method can then be used in all OECD member states	OECD Working Party of National Coordinators of the test guidelines programme (OECD-WNT)
Uptake in regulatory frameworks and use by stakeholders	In this phase regulatory bodies such as ECHA, EFSA and SCCS are involved to provide recommendation to implement the test guidelines into European policies. From then onwards these guidelines will be applied and can be used by stakeholders	European Commission (EC), European Chemicals Agency (ECHA) (chemical substances), European Food Safety Authority (EFSA) (food), Scientific Committee on Consumer Safety (SCCS)(cosmetics)

### The innovation system approach: key processes

In innovation system literature, seven key processes that are essential for the functioning of an innovation system have been identified. These key processes are knowledge development, knowledge diffusion, market formation, resource mobilisation, entrepreneurial experimentation, legitimacy creation and providing directionality (Hekkert et al 2007; Negro et al. 2007). Analysis of these key processes in the context of the implementation curve of NAMs shows that some processes align and overlap with the phases put forward by the implementation curve. These processes include knowledge development, knowledge diffusion, and market formation. The other key processes, i.e., resource mobilisation, entrepreneurial experimentation, legitimacy creation, and providing directionality, are not yet identified by the implementation curve, but are equally important for system performance (Hekkert et al. 2020; Elzinga et al. 2023) and as such for successful mission achievement towards animal-free safety assessment.

Aside from shedding light on how phases of the implementation curve are aligned with key processes and identifying additional processes not covered by the implementation curve, applying a systemic perspective furthermore emphasises that the key processes should not be conceived as linear and consequential, as might be implied by the implementation curve.^1^ Rather, as various NAMs are developed, diffused, and implemented in parallel and over time, processes constantly inform each other through interactions amongst actors involved (e.g., through coordination, cooperation, and resource exchange). Interactions between processes for instance take place when research to improve NAMs is translated into new requirements for NAM validation and implementation, and vice versa when regulatory needs and requirements provide direction for researchers in academia and companies to develop fit-for-purpose NAMs (Parish et al. 2020). It also has been stressed by toxicologists that these interactions are essential for efficient implementation and uptake of NAMs (Bos et al. 2020; Knight et al. 2021; Pistollato et al. 2021; Patterson et al. 2021). The innovation system approach stresses that interactions can take the form of bottlenecks and challenges that create negative feedback loops between processes and as such hinder the acceptance and uptake of innovations. Interactions can also create positive feedback loops by which processes interactively contribute to the uptake of NAMs (Hekkert et al. 2007; Suurs and Hekkert 2012).

The next section provides more detail on the key processes and how they relate to the phases described in the implementation curve.

### Integrating key processes for system performance and the NAM implementation curve

Table 2 describes the key processes for system performance and their relation to the implementation curve. It also gives the general description of each of the key processes from an innovation system perspective, based on Hekkert et al. (2007), as well as the interpretation of this general description in the form of activities specific to the development and uptake of NAMs for safety assessment.

**Table 2:** Key innovation system processes

Key processes	General description (based on Hekkert et al. 2007 and Elzinga et al. 2023)	Relation to implementation curve	NAM-specific description
Knowledge development	Knowledge production and research activities to develop innovations and advance them to higher Technology Readiness Levels (TRLs). This includes fundamental understanding of the natural world as well as design and construction activities to attain functional goals, which are supported by learning by doing. It also includes development of social and institutional knowledge on how to embed innovations in society	Research and development	Conducting research activities by universities, research institutes and companies to:
			Develop NAMs and improve design features and performance
			Create a better understanding of relevant underlying biological mechanisms using for example AOPs
			Contribute to understanding how to value NAMs for addressing risk problems and embedding NAMs in socio-institutional contexts
			Examples of research activities are conducted in the VHP4Safety project and the Animal-free Safety assessment of chemicals: project cluster for Implementation of novel Strategies (ASPIS) cluster projects
Knowledge diffusion	The diffusion of knowledge through scientific publications and other distribution channels such as professional journals, newspapers, conferences, flyers, campaigns etc. This includes knowledge management through open source development, open science and transdisciplinary research	Research and development	NAM databases (e.g., RE-PLACE, DB-ALM, NAT)
			Publishing of annual reports by for example EURL ECVAM with overview of NAMs and projects for NAM development (Zuang et al. 2023)
			Establishing networks for knowledge diffusion
			Stimulating use of FAIR principles for developing digital infrastructures for NAM data and creating repositories to share data on safety assessment
			Increasing involvement of stakeholders including regulators and NGOs in research projects from early phases of NAM development onwards
			Examples of communities are networks (international collaboration regulatory body in Accelerating Pace of chemical risk assessment (APCRA), the NAM working group of the International Liaison Group on Method for Risk Assessment of Chemicals in Food (ILMERAC), European Centre for Ecotoxicology and Toxicology of Chemicals (ECETOC), European Partnership for Alternative Approaches to Animal Testing (EPAA), conferences (e.g., EUSAAT, World Congress, on Alternatives to Animal Experimentation, ESTIV), journals (e.g., ALTEX)
Market formation	The formation and generalisation of (niche) markets for the use of innovative solutions. This includes stimulating these markets through for example subsidies, favourable regulation, in early stages as well as stimulating demand for practices and guidelines in order for solutions to become standardised	Validation, acceptance, uptake	Establishing a formal process for validation of NAMs towards formal regulatory acceptance in the guidelines of the OECD or EU. These formal processes help to provide signals to endorse by creating confidence and certainty for researchers, risk assessors, regulators, industry and CROs, in the system to use, diffuse and implement validated tests
	A key instrument is setting standards that can stimulate growth and demand of innovations, and can incite governmental agencies, academia and industry, to use and apply the innovations by creating certainty		Stimulating demand of practices and guidelines involves activities of ECHA, EFSA and SCCS, who provide recommendation and guidance about implementation of NAM-based test guidelines into European regulatory frameworks
Resource mobilisation	Mobilisation of human, financial and material resources to enable all other system processes. This includes mobilisation of competences and human capital through education in specific scientific and technological fields as well as in entrepreneurship, mobilisation of financial capital (seed and venture capital, diversifying firms, etc.) to make investments in the field and mobilisation of complementary physical assets such as complementary infrastructure, products and services	Not covered	Human resources: recruiting talented students, scientists and R&D personnel in universities (of applied science) and companies that have the knowledge to develop NAMs and advance them, facilitated by the development of (post)-graduate and professional teaching programmes and trainings on NAM development and use; regulators and policy-makers who are trained on how to interpret data and results generated by NAMs; governing actors who understand the complex governance structures in which NAM use and implementation is embedded. Examples include specific training programmes (e.g., Postgraduate Education in Toxicology (PET) course ‘NAMs for Toxicology’, ‘Evidence-based Toxicology’ by Center for Alternatives to Animal Testing (CAAT) at Johns Hopkins University, ETPLAS EU-52 and EU-56) funding programmes (e.g., Dutch Research Agenda (NWA) ‘Non-animal models: acceptance and implementation’ and EU H2020 SCI1-BHC-11–2020 ‘Advancing the safety assessment of chemicals without the use of animal testing’), physical resources (e.g., EURL ECVAM and EU-NETVAL labs)
			Financial resources: funding research projects and scaling-up of innovative solutions; investing in new lab facilities and/or software development; funding authorities to stimulate regulatory innovation and experimentations with NAMs
			Physical resources: creating shared infrastructure/laboratory equipment and facilities for the development, validation and implementation of NAMs
Entrepreneurial experimentation	Entrepreneurs, researchers and pioneers that dare to take risks and experiment in order to demonstrate the value of innovations. This includes entrepreneurs who enter new markets with innovative solutions and pursue business model innovation to promote the diffusion of these innovations	Not covered	Taking a risk as a company to develop new NAMs
			Experimenting as governmental actor with new protocols and guidance documents established during expert workshops including the development of new principles and criteria for validation and regulatory acceptance of new and revised test methods Examples include multiple stakeholder initiatives (e.g., European Organ-on-Chip Society (EUROoCS) towards standardisation of organ-on-chips) and expert workshops (e.g., ECHA NAM workshop and the Workshop on the European Commission roadmap towards phasing out animal testing for chemical safety assessments) or the OECD IATA Case Studies Project
Legitimacy creation	Reducing resistance to change and increasing trust, support and societal acceptance of innovations through lobby activities, advocacy coalitions and knowledge campaigns	Not covered	Lobbying/campaigning towards end-users highlighting opportunities of NAMs and limitations of animal testing
			Put pressure on governments to reduce or ban animal-testing
			For example, the European Citizens' Initiative (ECI) ‘Save Cruelty-free Cosmetics—commit to a Europe without Animal Testing'
Providing directionality	Formulating expectations and visions as well as the translation of these visions into mission objectives, policy goals and regulatory requirements. This includes actions aimed at coordinating actors, resources, and institutions to contribute and align around set objectives	Not covered	Creating a level of agreement on the urgency of the problem of animal testing versus other problems in environmental and health risk regulation as well as which specific problems (e.g., toxicological endpoints, substance classes) to prioritise. An example is the New Approach Methodologies Workshop: towards an Animal Free Regulatory System for Industrial Chemicals organised by ECHA (2023), where the challenges of NAM-based testing and at the same time ensuring safety of industrial chemicals were addressed
			Aligning expectations on what type of solutions should be supported or not (e.g., complete animal-free versus partial use, like lower species and animal tissues)
			Articulating expectations and visions regarding (specific classes of) NAMs and their future context of use. Examples include visions on application of NAMs in next generation risk assessment, described in roadmaps such as the EFSA NAM Roadmap (Escher et al. 2022), and endorsed in high-level guidance documents on for example integration of different NAMs, such as the OECD guidance document on IATAs (OECD 2020)
			Bringing actors together to mutually formulate missions and roadmaps such as those mentioned in Box 1 (see supplementary file) and outline how such missions can be translated into policy and regulatory change
			Creating networks and coalitions for knowledge exchange and goal setting in which actors involved in different key processes in the system come together (e.g., the European Partnership for Alternative Approaches to Animal testing (EPAA) and the Netherlands Transition Programme towards animal-free Innovation (TPI)
			Initiating and aligning missions and roadmaps developed at multiple levels (e.g., European, national, regional) as well as for different application contexts (e.g., chemical safety assessment, regulatory nonclinical safety assessment of drugs, use of animals in fundamental scientific research)

#### Key processes that overlap the implementation curve

The first phase of the implementation curve, known as the research and development (R&D) phase, describes activities that are captured by the key process *knowledge development*. This entails the development of NAMs, including knowledge on regulatory relevance and biological and chemical applicability domain, often performed in collaborative research projects and case studies. In addition to knowledge development, the innovation system approach explicitly adds the notion of *knowledge diffusion* to which the R&D phase aligns, to facilitate the uptake of new knowledge, methods, mechanisms of toxicity, et cetera through dissemination channels, including scientific publications, databases, workshops, expert groups, or conferences. In the context of NAM development, a wide range of actors that participate in the research projects and case studies already contributes to this dissemination, including universities, research institutes, small- to large-sized companies, funders and non-governmental organisations (NGOs). These actors collectively help to disseminate knowledge, stimulate learning and create spillovers to actors in the system.

The validation, acceptance, and uptake phases of the implementation curve align with the key process *formation of a market*. As NAMs go through the process of validation, acceptance, and uptake, involved organisations and regulatory actors contribute to forming demand and a market for their use. After uptake, NAMs can be legitimately used for dossier-building and support regulatory decision-making, further contributing to market formation. Increased demands for NAMs can subsequently destabilise the traditional demands for animal testing and consequently provide room for safety testing without using animals.

#### Key processes that complement the implementation curve

Next to the key processes that overlap the implementation curve, this section details four additional key processes described in the innovation system approach (in *italics*) that complement it. Activities relevant for NAM development and uptake within these processes (underlined) are also described.

First, the additional key process *resource mobilisation* is relevant in all phases of the implementation curve, from R&D to implementation. Specifically, there are three kinds of resources: human capital, and physical and financial resources. When it comes to human capital, an important activity is recruiting and educating talented researchers, risk assessors, and regulators. Researchers that have the knowledge to develop NAMs for safety assessment are crucial in the R&D phase. When it comes to the validation and acceptance phase, there is subsequently a need for researchers and risk assessors who understand the methodology and results of NAMs and how to interpret them in the regulatory context of use, and evaluate them accordingly. In the implementation phase, there is a need for regulators that know how to incorporate NAMs in regulatory frameworks on national and international levels. New research centres like the Centre for Animal-free Biomedical Translation (CPBT) may focus on developing educational and training programmes required to train actors on specific needs to stimulate progression of NAMs from one phase to another towards their ultimate uptake into regulatory protocols. Additionally, throughout all phases, securing financial resources is needed to not only fund research activities but also validation studies, helping NAMs forward towards implementation into regulatory frameworks. Finally, securing physical resources, for example necessary when constructing new research centres like the CPBT in the Netherlands (RIVM 2024) or platforms for validation such as PEPPER, is important in this key process. Examples of these resources can be found in Table 2.

Second, *entrepreneurial experimentation* is a key process that aligns with the R&D and validation phase. In addition to experimentation in the R&D phase—to which actors such as universities, universities of applied science, and R&D departments in industry and research institutes contribute—this key process includes engaging in business model innovations to foster the uptake of NAMs. This type of entrepreneurial experimentation includes the innovative capacity and efforts by industry actors from small- to large-sized companies to develop NAMs. They experiment with their use in-house or provide it as a service to other companies, even when these methods are not yet accepted for regulatory use. NAMs developed in this context could be submitted as test methods to start validation studies. The organ-on-a-chip technology is an example where an advanced market is formed including specialised companies and standards development (Zhang and Radisic 2017; Piergiovanni et al. 2021).

Third, the key process *legitimacy creation* allows for prioritising NAMs, at the cost of other practices and technologies based on animal testing for safety assessment. This process entails increasing the trust in and support for and societal acceptance of NAMs and at the same time reducing the resistance to change. Creating social legitimacy includes facilitating a cognitive change in belief systems amongst relevant actors in the system towards the acceptance and use of NAMs. In addition to industry, academia, and governmental actors, a wide array of actors contributes to this process such as NGOs (e.g., animal rights, environmental sustainability, patient representation groups, and consumer rights) trade societies and civil society in general. Together, NGOs might promote animal welfare as well as human safety and health. The activities revolve around reconfiguring belief systems that central actors in safety assessment adhere to (e.g., from a belief system where the animal study is considered as the gold standard (Sewell et al. 2017; Westmoreland et al. 2022) towards a belief system in which NAMs provide support for human health safety assessment). Lobbying and campaigning towards end-users and building trust towards acceptance of NAMs are also an important part of this process (Wassenaar et al. 2024). One way to achieve this is by discrediting current testing methods using animals by, for example, disconnecting the moral foundations of particular norms and undermining taken-for-granted assumptions (e.g.,) “using animals for safety testing is the safest and most reliable method”). Researchers have, for example, used a systematic approach of outlining uncertainties and limitations of animal testing for certain endpoints (Paparella et al. 2017, 2020), or outlined fundamental principles such as robustness and benefit to benchmark animal testing for specific contexts of use (Pallocca et al. 2022).

A fourth and final additional key process that the innovation system approach describes is that of *providing directionality*. This process describes and explains the importance of governance in innovation processes. Governance includes formulating expectations and visions as well as the translation of these visions into mission objectives. An important activity within this process is formulating policy goals, regulatory requirements and mission statements. In general, providing directionality is strongly tied to policies and regulation for chemical testing and the governance of regulatory authorities, such as ECHA and EFSA. In this context, acceptance of NAMs can be stimulated by formulating measurable, ambitious, and timebound mission objectives. Formulating and sticking to mission objectives create certainty and perspective for actors that aim to invest in the development, validation, and uptake of NAMs. It also provides a clear frame for specifying short-term goals focused on for instance progressing NAMs from research and development to uptake or changing regulatory requirements. Providing directionality entails that relatively powerful actors need to fulfil a coordinating role in structuring, shaping, and mobilising other actors, resources, and institutions to contribute and align around set mission objectives. While this role can be fulfilled by any type of actor or a consortium of actors, governmental actors are expected to play a key role due to the highly regulated environment in which NAMs need to be implemented as well as the need for harmonising requirements across jurisdictions. Recent NAM-specific initiatives in the context of providing directionality are the ECHA New Approach Methodologies Workshop (ECHA 2023) and the European Commission roadmap towards phasing out animal testing for chemical safety assessments (European Commission 2023a; b). What is very interesting about these initiatives is the organising actor. The fact that governmental institutions, such as ECHA, take responsibility and spend resources to lead these initiatives, broadens the ownership of the topic, which in itself can already cause acceleration.

In the context of the implementation curve, providing directionality also entails that policy-makers and other actors express expectations and visions about the promise of a certain NAM or NAM-based approach. This takes shape in, for example, visions on next generation risk assessment (EPA 2018) and toxicity testing for the twenty-first century (National Academies 2007). It also requires work of actors on guidance documents that facilitate the integration of NAMs for addressing regulatory questions such as with the Guidance Document on Good In Vitro Method Practices (OECD 2018) or the concept of and guidance for Integrated Approaches to Testing and Assessment (IATA) (OECD 2020). Providing directionality is also visible in how to define NAMs (e.g., New Approach Methodologies versus Non Animal Methods) in the first place and whether explicit references to non-animal methods or non-vertebrate animals are made in this context.

## A comprehensive framework to analyse progress towards an animal-free chemical safety assessment system

**Fig. 2:**
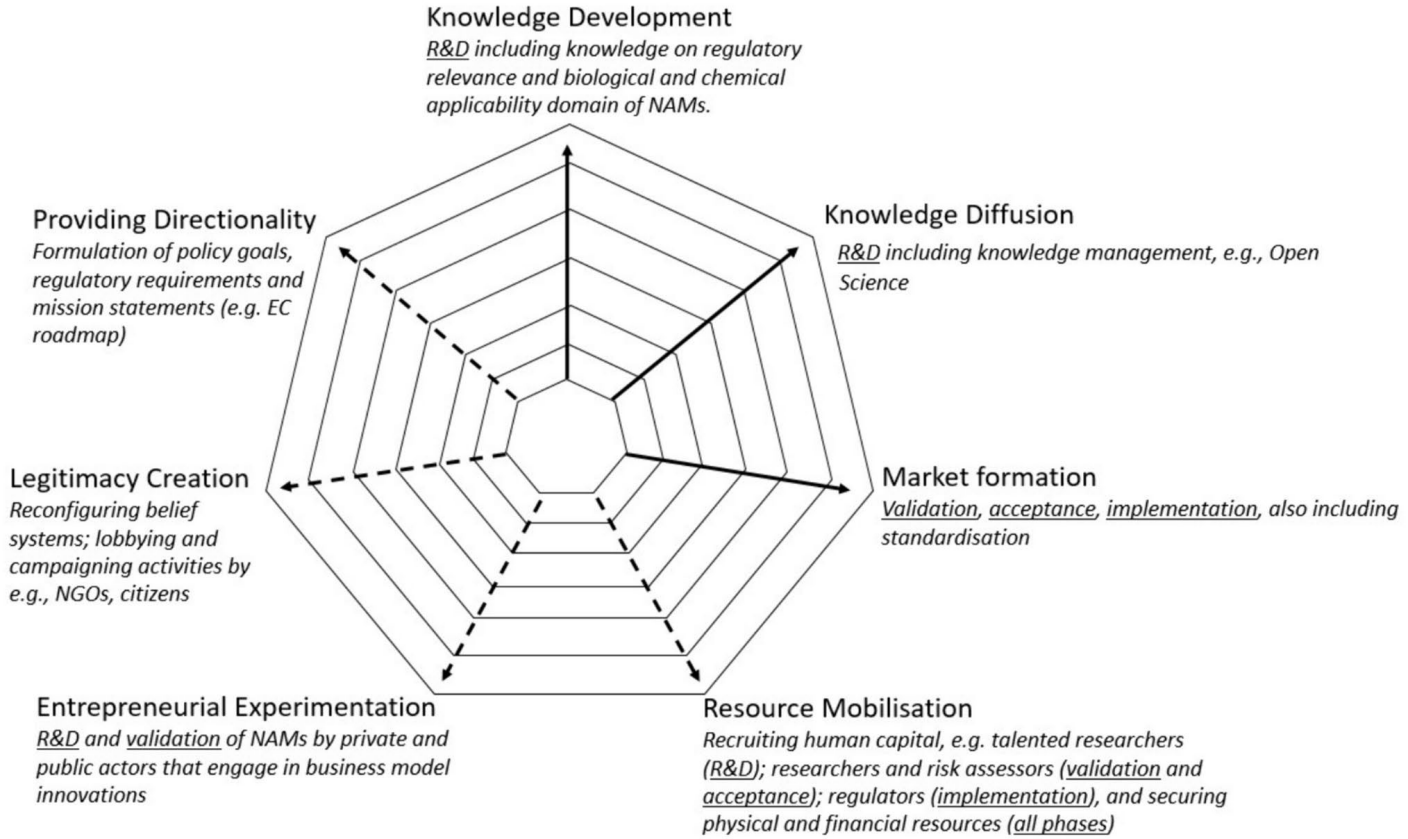
The integration of key processes and phases (underlined) in the NAM implementation curve. The dotted lines indicate the key processes that elucidate complementary activities for the implementation curve

Based on the integration of insights offered by both the implementation curve as developed by toxicologists, and the innovation system approach as developed by innovation scholars, we constructed a comprehensive framework that offers a way to analyse the progress towards a novel system based on animal-free safety assessment of chemicals. Figure 2 presents this framework as a radar diagram showing the interconnection of the key processes derived from the innovation system approach and the phases from the implementation curve. It also presents relevant activities per key process and emphasises that key processes can interact with each other to incite or hamper the mission towards animal-free innovations.

The arrows represent levels that can be scored in a qualitative manner when assessing the performance of the key processes i.e., a functional analysis. The dotted arrows indicate the four additional key processes that are complementary to processes covered by the implementation curve. These complementary key processes shed light on important activities that need to be considered in the mission towards the implementation of NAMs in addition to R&D and market development: *resource mobilisation* emphasises the importance of recruiting talented researchers, risk assessors, and regulators that have the appropriate knowledge to help NAMs progress, as well as securing financial and physical resources. *Entrepreneurial experimentation* emphasises the importance of private and public actors engaging in business model innovations. *Legitimacy creation* emphasises the importance of reconfiguring belief systems as well as lobbying and campaigning activities by for instance NGOs. Finally, *providing directionality* emphasises the importance of formulating mission statements, policy goals, and regulatory requirements.

## Discussion: applying the innovation system perspective to accelerate the mission towards animal-free safety assessment

In an interdisciplinary effort with toxicologists and innovation scientists, we have introduced the innovation system approach to understand what is needed for mission achievement towards animal-free safety assessment against a background of slow uptake of NAMs in regulatory frameworks. Complementary to the implementation curve approach (RIVM 2022), the innovation system approach (Hekkert et al. 2007; Elzinga et al. 2023) stretches beyond understanding individual processes of, e.g., research and development and acceptance of NAMs. It creates awareness that the transition to animal-free safety assessment with the required uptake of NAMs in regulatory frameworks is dependent on creating positive feedback loops between interconnected processes. As such, it should not be seen as a linear, consequential process of NAM implementation. It shows how a broad range of activities, conditions, and actors shape innovation processes through seven interconnected key processes. Providing insight into the functioning of these seven processes is generally based on two analyses: a structural analysis and a functional analysis.

A structural analysis of the innovation system comprises the mapping of all relevant actors and institutions involved in individual key processes or combinations of them, as well as their interrelations, roles, and responsibilities. Obtaining these insights can raise awareness amongst actors of their role, whether all relevant actors and institutions are present or not, and provide insight into how these actors can contribute to shaping key processes in the innovation system by performing relevant activities. Future research employing this type of analysis of the structure of the innovation system can contribute to identifying incumbent and new actors as well as the key activities they employ to drive and accelerate mission achievement towards animal-free safety assessment.

In addition, functional analysis of the innovation system consists of an assessment of how key processes relevant for effective use of NAMs contribute to mission achievement both individually and in interaction with each other. Functional analysis can be forward-looking (formative) to help to understand why and how system performance and missions are expected to work (or not) as well as backward-looking (summative) which serve to attribute observed outcomes to the effects of governance efforts (Janssen et al. 2022). In both cases, the analysist can focus on scoring the presence and functioning of individual key processes (by assessing the quantity and/or quality of activity exerted in relation to the key process) to identify where efforts are lacking or needed. Identification of such intervention points can then inform further discussions on concrete (sets of) interventions to stimulate innovation system performance.

From a systemic perspective, functional analysis also comprises the identification of drivers that shape positive feedback loops as well as barriers that shape negative feedback loops in the innovation system (Suurs and Hekkert 2012). Interesting avenues for this type of analysis are, for instance, to study how efforts relating to *providing directionality* (e.g. the EC roadmap) can have a positive influence on *resource mobilisation* which then contributes to *research and development* efforts including the validation of NAMs, which then in turn reinforces the set direction as part of mission achievement (positive feedback loop). Alternatively, a lack of *legitimacy creation* (e.g., the ambition of the Netherlands to be world leader in animal-free innovations by 2025 as stated in a letter to the parliament in 2016 (van Dam 2016), was later toned down towards the Netherlands as a forerunner in this field and further loosened up by letting go of the year 2025 (Schouten 2018)) can limit trust in NAMs which hampers industry and other actors to engage in *entrepreneurial experimentation* activities resulting in deadlock situations (negative feedback loop).

In conclusion, we argue that more attention needs to be paid to the systemic nature of challenges relating to NAM development and implementation. The NAM implementation curve describes the more expert driven, scientific and technical changes towards acceptance and effective use of NAMs and animal-free safety assessment of chemicals. The innovation system approach provides a broader perspective, revealing key processes at a more societal level that need to be enhanced for successful implementation of NAMs. Furthermore, acknowledging the interconnection between key processes allows to reveal positive and negative feedback loops that either drive or hamper the transition. The structural and functional analysis we suggest in this innovation system approach can focus on particular domains (e.g., cosmetics, food, industrial chemicals, and pharmaceutical products), geographical contexts (e.g., European Union, United States), and safety assessment contexts (e.g., human health versus environment), and will provide much needed insight into systemic intervention points, prioritisation, and strategies for technological, societal, and institutional changes to accelerate acceptance and effective use of NAMs to realise the mission of animal-free safety assessment.

## Supplementary Information

Below is the link to the electronic supplementary material.

Supplementary file1 (DOCX 15 KB)

## Data Availability

No datasets were generated or analysed during this study.

## Footnotes

1 Importantly, the non-linear nature is also illustrated by NAMs such as physiologically based kinetic (PBK) models and biomonitoring tools, which did not pass through all the implementation phases before being used in a regulatory context. Additionally, in recent discussions, changes were proposed in standard information requirements in the revision of the REACH Regulation (EC No 1907/2006), including NAMs that have not fully completed the implementation curve (Schutte 2020).
