# Supplementary material for: Animal-free safety assessment of chemicals: an innovation system perspective
Source: Arch Toxicol. 2024 Oct 4;99(1):43–56. doi: 10.1007/s00204-024-03878-0 (PMC11742464; doi:10.1007/s00204-024-03878-0)
Supplement: Supplementary file 1 — Supplementary file1 (DOCX 15 KB) [file 204_2024_3878_MOESM1_ESM.docx]

**Supplementary files**

Box 1: Missions and roadmaps towards animal-free safety assessment of chemicals

Examples of mission statements and roadmaps have been formulated by several national and international organisations and authorities. Amongst these is the RIVM roadmap, published in 2018, for animal-free innovations in regulatory safety assessment. The RIVM roadmap identified steps that need to be taken towards a future without the use of animals for safety assessment. Various national and international stakeholders from government, scientific research and industry were involved in the development of the roadmap and emphasised that on the short term, it is not yet feasible to fulfil the requirements for safety assessment in the regulatory frameworks without using animals (RIVM, 2018).

Also in 2018, the Interagency Coordinating Committee on the Validation of Alternative Methods (ICCVAM) published a strategic roadmap for establishing new approaches to evaluate the safety of chemicals and medical products in the United States. The ICCVAM roadmap presented plans for implementation of methods to eliminate or reduce animal testing in toxicology. These plans included the establishment of working groups on technology, confidence and utilisation of new approaches and is currently further elaborated to provide insights on building confidence in NAMs (ICCVAM, 2018; 2024).

More recently, examples of strategies include the European Food Safety Authority (EFSA) strategy 2027, which includes a strategic objective on ensuring preparedness for future risk analysis needs. One of the expected outcomes formulated by EFSA on this objective is to improve the quality of scientific guidance and methodologies and to address future challenges including the development and integration of NAMs and the minimisation of animal testing (EFSA, 2021). This EFSA strategic objective was addressed in a roadmap for action on New Approach Methodologies, the outcome of an EFSA procurement titled Development of a Roadmap for Action on New Approach Methodologies in Risk Assessment-lot 2 (reference number OC/EFSA/ED/2020/01). In this roadmap, experts advised EFSA “on a multi-annual strategy for increasing the use of NAMs in EFSA human health risk assessment to minimise the need for animal-based verification studies, so that by 2027, the large majority of EFSA requests for additional data are based on integrated approaches using NAMs” (Escher et al., 2022).

In 2023, the European Commission (EC) responded to the European citizens’ initiative on strengthening the cosmetics animal testing ban, transforming chemical regulations, and modernising science through legislative changes. The EC response included to “kick-off work on a roadmap towards replacing animal testing in chemical safety assessments” and organising a workshop on the Commission roadmap towards phasing out animal testing for chemical safety assessments (European Commission, 2023a; 2023b).
